# Evidence for Tautomerisation of Glutamine in BLUF Blue Light Receptors by Vibrational Spectroscopy and Computational Chemistry

**DOI:** 10.1038/srep22669

**Published:** 2016-03-07

**Authors:** Tatiana Domratcheva, Elisabeth Hartmann, Ilme Schlichting, Tilman Kottke

**Affiliations:** 1 Department of Biomolecular Mechanisms, Max Planck Institute for Medical Research, Jahnstraße 29, 69120, Heidelberg, Germany; 2 Physical and Biophysical Chemistry, Department of Chemistry, Bielefeld University, Universitäts straße 25, 33615 Bielefeld, Germany

## Abstract

BLUF (blue light sensor using flavin) domains regulate the activity of various enzymatic effector domains in bacteria and euglenids. BLUF features a unique photoactivation through restructuring of the hydrogen-bonding network as opposed to a redox reaction or an isomerization of the chromophore. A conserved glutamine residue close to the flavin chromophore plays a central role in the light response, but the underlying modification is still unclear. We labelled this glutamine with ^15^N in two representative BLUF domains and performed time-resolved infrared double difference spectroscopy. The assignment of the signals was conducted by extensive quantum chemical calculations on large models with 187 atoms reproducing the UV-vis and infrared signatures of BLUF photoactivation. In the dark state, the comparatively low frequency of 1,667 cm^−1^ is assigned to the glutamine C=O accepting a hydrogen bond from tyrosine. In the light state, the signature of a tautomerised glutamine was extracted with the C=N stretch at ~1,691 cm^−1^ exhibiting the characteristic strong downshift by ^15^N labelling. Moreover, an indirect isotope effect on the flavin C_4_=O stretch was found. We conclude that photoactivation of the BLUF receptor does not only involve a rearrangement of hydrogen bonds but includes a change in covalent bonds of the protein.

Photoreceptor proteins mediate responses in living organisms by adopting two distinct structural conformations depending on the light conditions − the dark state and the light state. Switching between the two states occurs upon the photochemical modification of the chromophore cofactor bound to the receptor protein. The numerous photoreceptor proteins known today display a diversity of light-sensing cofactors that typically undergo double-bond isomerization or redox chemistry^1^,^2^.

The blue light sensor using flavin (BLUF)^3^ is a photosensory protein domain with a unique feature of preserving the chemical structure and, therefore, the UV-vis spectrum of its flavin chromophore in the two functional states^4^,^5^. Photoreceptor proteins containing the BLUF domain control gene expression or secondary messenger metabolism in bacteria and in the eukaryote *Euglena gracilis*^5^,^6^,^7^,^8^. All characterised BLUF domains demonstrate only a small red shift of the flavin absorbance maximum at around 450 nm upon photoexcitation, indicating some changes induced in the local environment of the chromophore. Concomitant with the red-shifted flavin absorption, a pronounced downshift of the flavin C_4_=O stretching frequency was identified, indicating a strengthening of the hydrogen bonding with the protein in the light state^9^. On the second to minutes time scale, depending on the particular BLUF protein, the red-shifted state undergoes thermal conversion back to the dark state.

Initial interpretation of these light-induced spectral shifts was attempted on the basis of the crystal structures. The two BLUF protein conformations identified in the crystal structures^10^,^11^,^12^,^13^,^14^,^15^, termed Met-in and Trp-in, differ in the folding of the C-terminus and in the positioning of either methionine or tryptophan inside the flavin binding pocket. Additionally, the conformations differ in the orientation of the conserved glutamine amide group, which is rotated by 180 degrees. This rotation of the glutamine side chain upon photoexcitation was proposed to cause the observed red shifts of the UV-vis and infrared spectra^16^,^17^,^18^. As an alternative mechanism to rationalise the red shifts, it was suggested that the glutamine might undergo a tautomerisation to the Z-Z imidic acid^19^,^20^,^21^. Altogether four opposing chemical mechanisms of the BLUF photoactivation reaction via glutamine modifications are discussed in the literature as resulting from these considerations (Fig. 1). The key role of the glutamine was confirmed by spectroscopy and kinetic experiments in combination with mutagenesis^19^,^22^,^23^,^24^,^25^, which however did not provide sufficient data to unambiguously identify the glutamine orientation in the dark state and its light-induced modification. Moreover, the results of quantum chemical calculations linking experimental and theoretical spectra to reveal the glutamine structure in the dark and light states provided support for the opposing glutamine rotation and tautomerisation photoreactions^18^,^20^,^21^,^26^,^27^,^28^,^29^.

Obtaining experimental evidence for the presence or the absence of a tautomerisation of the conserved glutamine would constitute the most straightforward way to clarify the BLUF photoreaction. Infrared spectroscopy in conjunction with ^15^N labelling of the glutamine side chain provides a suitable approach for distinguishing between the C=N and C=O bonds. Even if all glutamines of BLUF contain the ^15^N label because of experimental limitations, only the glutamine positioned in the flavin binding site might show the specific isotopic shift of the tautomerisation upon photoactivation. The effect of the hydrogen bonds on isotopic shifts should be evaluated by quantum chemical calculations of sufficiently large active site models representing the Met-in and Trp-in conformations of a BLUF domain.

We introduced ^15^N-labelled glutamines in two BLUF proteins, BlrB from *Rhodobacter sphaeroides* as a short BLUF protein^13^ and the BlrP1-BLUF domain from *Klebsiella pneumoniae*, which is part of a light-regulated phosphodiesterase^11^. Using a combined infrared spectroscopic and quantum chemistry approach, we provide evidence for the structure and interactions of the glutamine in the dark state, which are consistent with the Met-in orientation in the crystal structure. Moreover, we identified the spectral signature of the glutamine imidic acid tautomer in the light state.

## Results

### Principle behind the glutamine labelling approach

We selected ^15^N labelling at the ε-position as a suitable approach to distinguish between rotation and tautomerisation of the glutamine side chain. Calculations of the infrared spectra of the model compounds acetamide and its Z-Z imidic acid tautomer illustrate our approach (Fig. 2). In the central region of carbonyl vibrations of 1,600–1,800 cm^−1^, unlabelled acetamide shows a C=O stretch frequency determined by the extent of hydrogen bonding. Upon ^15^N labelling, this frequency undergoes a small downshift by 1 cm^−1^ because of the coupling with the NH_2_ bend (Fig. 2a). The latter vibration is found below 1,600 cm^−1^ and is downshifted by 8 cm^−1^ upon labelling. In contrast, the imidic acid tautomer shows a C=N stretch at above 1,600 cm^−1^, which undergoes a 16-cm^−1^ downshift by ^15^N labelling (Fig. 2b). The relative position of the C=O and C=N stretches can vary depending on the influence of the hydrogen bonding and polar environment (Fig. 2c,d) whereas the small ^15^N isotopic shift of the C=O stretch and the pronounced ^15^N isotopic shift of the C=N stretch are almost invariant. Therefore, formation of the imidic acid of glutamine in the BLUF domain can in principle be detected experimentally by the strong effect of the specific ^15^N isotopic labelling.

### FTIR spectroscopy on BlrB and BlrP1-BLUF

The short BLUF domain protein BlrB was labelled at up to 75% yield with ^15^N at the ε-position of all glutamines. The infrared difference spectrum of BlrB was generated by recording spectra directly before and after illumination with blue light using the time-resolved rapid-scan technique (Fig. 3a). In the difference spectrum, only those vibrational bands are detected that change in frequency or intensity upon illumination. Negative bands originate from the dark state of the BLUF domain, whereas positive bands correspond to the light state. For direct comparison, unlabelled BlrB was prepared and investigated (Fig. 3a). The difference spectrum of unlabelled BlrB was scaled to that of ^15^N-Gln BlrB without any bias by calculating a least-squares minimization of deviations in the range 1,500–1,200 cm^−1^. In this spectral range, only very minor effects are evident from labelling.

In the difference spectrum, the prominent band pair at 1,710(−)/1,695(+) cm^−1^ includes strong contributions from the C_4_=O stretch of flavin with an amide I band adding to the positive signal^30^. The bands at 1,641(−)/1,623(+) cm^−1^ are tentatively assigned to amide I contributions by comparison with AppA-BLUF^30^. Isotopic labelling of glutamines introduces minor but distinct changes in the spectrum. Most changes are far more prominent than the variation from sample to sample (Supplementary Fig. S1). Only at 1,580–1,550 cm^−1^, the variation from sample to sample is equivalent to the signal most likely because of a spectrometer drift, which prevents any evaluation of this spectral region. The spectral congestion between 1,700–1,600 cm^−1^ does not allow to directly isolate difference bands of the C=O stretches that might contribute to this spectral region. A closer inspection reveals that two major shifts of absorbance are caused by labelling, which lead to intersections at 1,693 and 1,664 cm^−1^, respectively (Fig. 3a, inset).

BLUF domains trigger many different functions in a variety of organisms. Therefore, it was necessary to verify whether the changes in the vibrational pattern of BlrB by labelling are reproducible in other BLUF domains and thereby can be generalised. The BlrP1-BLUF was chosen for comparison and subjected to the same labelling procedure. Difference spectra of BlrP1-BLUF show a pronounced difference in intensities compared to those of BlrB in the spectral range of the amide I bands (1,615–1,695 cm^−1^) indicating significant differences in the secondary structural changes upon illumination (Fig. 3b). The spectra of BlrB and BlrP1-BLUF were scaled on the flavin C_4_=O stretch at 1710(−) and 1707(−) cm^−1^, respectively, and the integral intensity of the bands in the amide I region was calculated under the assumption of a homogeneous decay of all bands after illumination (Supplementary Fig. S2). It can be concluded that BlrP1-BLUF undergoes significantly smaller conformational changes compared to BlrB. In contrast, the frequency of the C_4_=O stretch of flavin in its dark form at 1,707 cm^−1^ shows only a minor difference of 3 cm^−1^ in the direct comparison of the two BLUF domains, which points to a similar flavin binding pocket in line with their crystal structures^11^,^13^. Difference spectra are almost identical for unlabelled and ^15^N-Gln BlrP1-BLUF. This similarity can be attributed to the much lower degree of incorporation of the ^15^N isotopomer in the BlrP1-BLUF sample of ~58% compared to ~75% for the BlrB sample.

To illustrate the spectral changes caused by labelling, a double difference spectrum of the difference spectra of unlabelled BlrB *minus*^15^N-Gln BlrB was calculated (Fig. 4a). In the double difference spectrum, band pairs are observed at 1,697(+)/1,689(−) and 1,667(−)/1,660(+) cm^−1^. Moreover, a minor, third difference band at 1,713(−)/1,708(+) is resolved. It should be noted that a strong difference band is observed only in the double difference spectrum for a significant labelling-induced frequency shift. Otherwise the difference bands of the unlabelled sample and of the ^15^N isotopomer cancel each other in the double difference. In general, positive bands in the double difference spectrum can originate either from the light state of the unlabelled sample or from the dark state of the labelled sample and vice versa for the negative bands. However, it needs additionally to be taken into account that isotopic labelling results in a downshift of a vibrational band because of increase in mass. From this fact it can be concluded directly that the difference band at 1,697(+)/1,689(−) cm^−1^ originates from a vibrational mode of the light state and its labelled counterpart, whereas the other two difference bands correspond to signals of the dark state and their downshift in frequency upon labelling.

The corresponding double difference spectrum was also calculated for the BlrP1-BLUF domain revealing a similar pattern to that of BlrB (Fig. 4a). Evidently, both BLUF domains show the same signature of light- and labelling-induced differences albeit slightly upshifted by 3–8 cm^−1^ in BlrP1-BLUF compared to BlrB. This similarity is taken as an approval of the generality and reproducibility of these signals for the BLUF domain photoreceptors.

**Figure 1:**
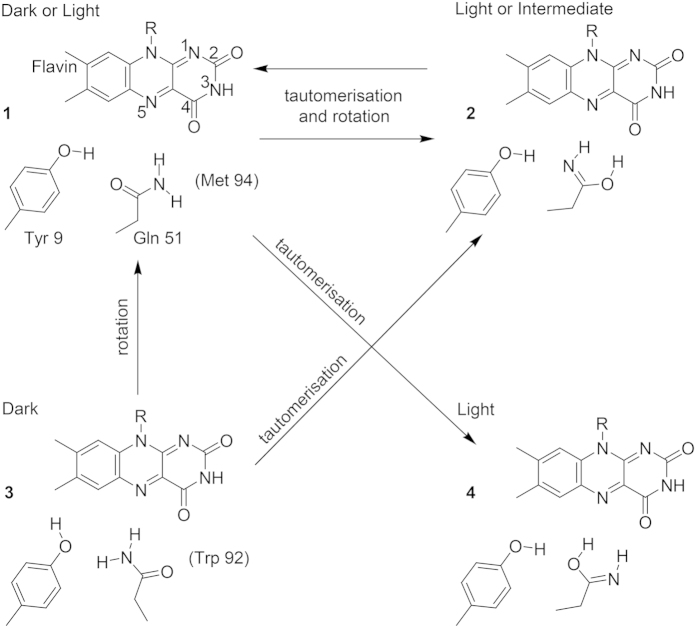
Chemical structures of the dark and light states in BLUF photoreceptors. The arrows indicate the proposed photoactivation reactions of the conserved glutamine: **3** → **1** rotation^16^−^18^; **1** → **2** tautomerisation to the Z-Z imidic acid and rotation^20^; **1** → **4** tautomerisation to the Z-Z imidic acid^21^; **3** → **2** → **1** rotation via transient tautomerisation^19^,^23^. Structures **1** and **3** show glutamine orientations in the Met-in and Trp-in structures, respectively. Residue numbering is according to the BlrB sequence. Numbering of the flavin atoms is indicated in structure **1**.

**Figure 2:**
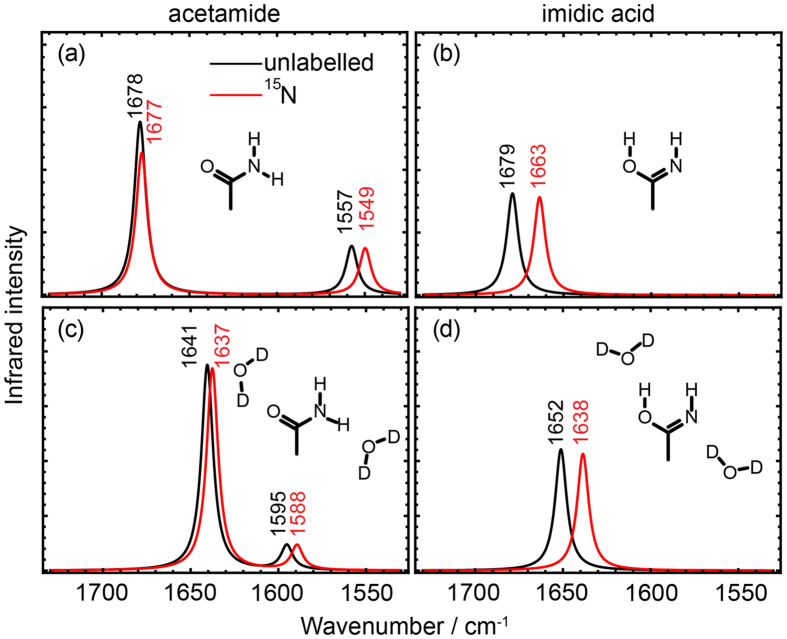
Effect of ^15^N labelling for distinguishing the amide and imidic acid tautomers. Infrared spectra were computed of the amide and Z-Z imidic acid forms of acetamide in bulk water (panels (**a**,**b**)), and in combined molecular (two D_2_O molecules) and bulk water (panels (**c**,**d**)). The chemical structures of the computed molecules are included in the figures. Results for unlabelled tautomers are shown in black, for ^15^N-labelled tautomers in red. Tautomerisation to the imidic acid is distinguishable by a prominent 14-cm^−1^ downshift of the C=N stretch by ^15^N labelling in the spectral region of 1,650–1,700 cm^−1^.

**Figure 3:**
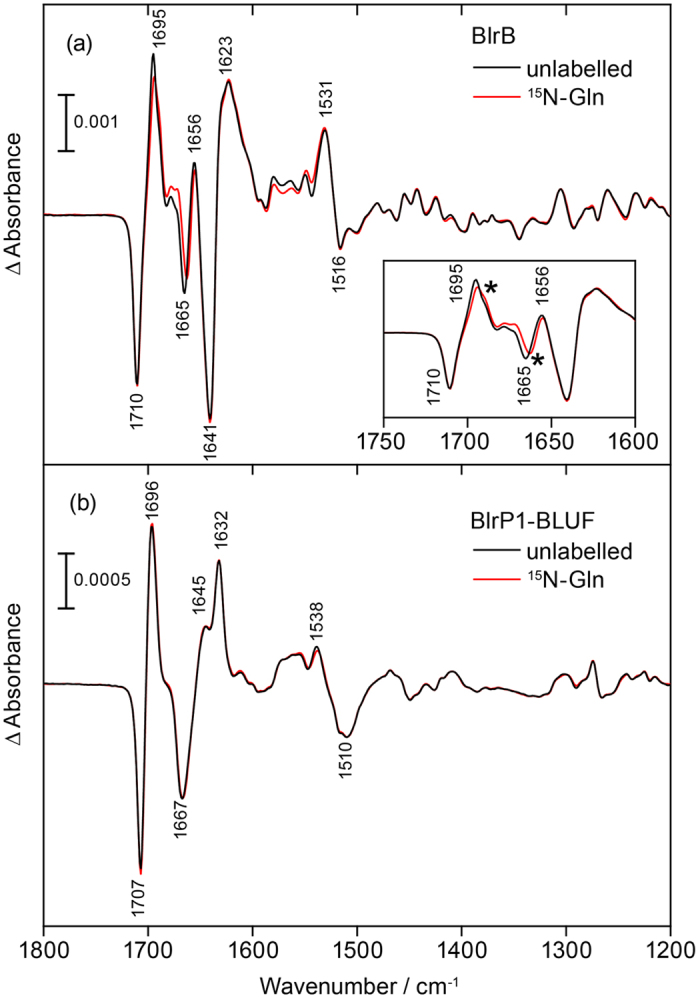
Light-induced FTIR difference spectra of two BLUF domains with and without ^15^N labelling of glutamines. (**a**) The difference spectra of BlrB show positive bands originating from the light state and negative signals from the dark state. Inset: The close-up view of the difference spectra reveals clear shifts of absorbance between labelled (red) and unlabelled (black) BlrB, which lead to two intersections depicted with an asterisk. (**b**) The difference spectra of labelled (red) and unlabelled (black) BlrP1-BLUF are similar because of the low isotopomer ratio achieved. The difference spectra differ significantly from those of BlrB especially in the frequency region of secondary structural changes at around 1,650 cm^−1^.

**Figure 4:**
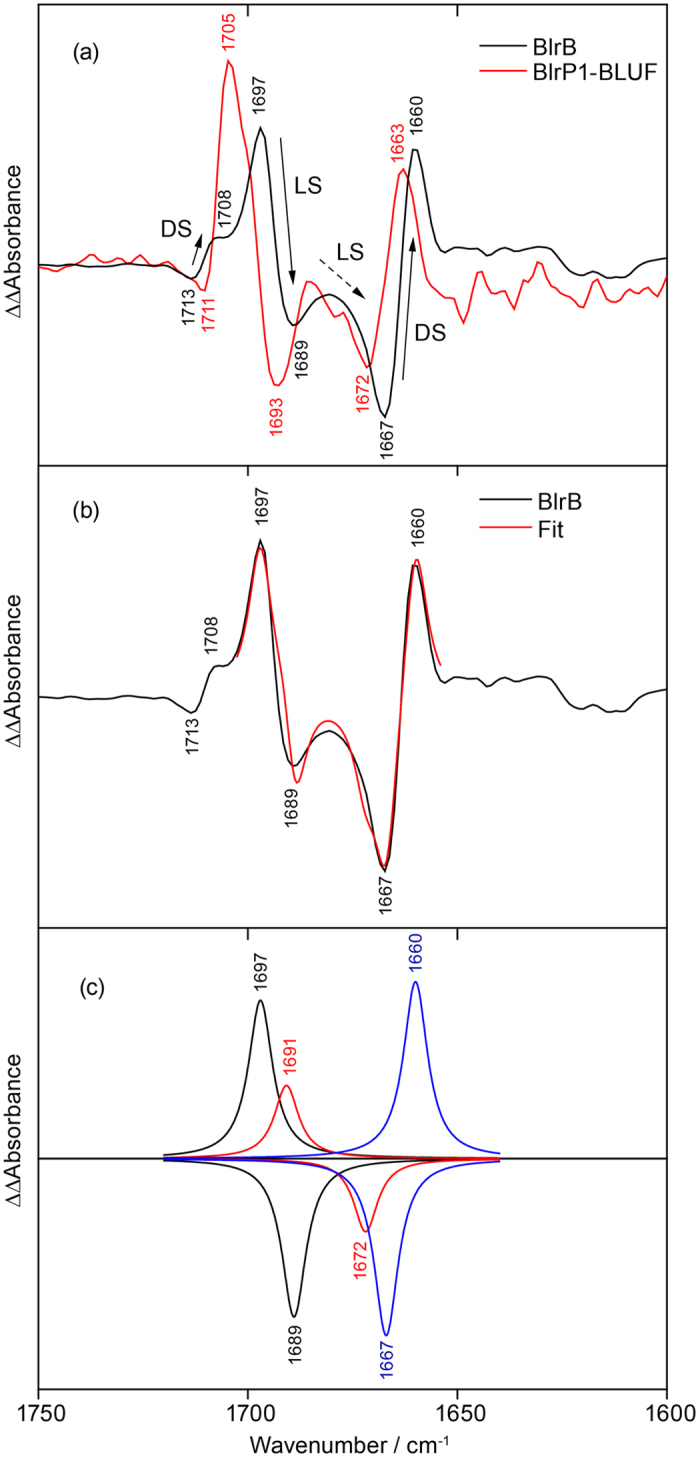
Double difference spectra of unlabelled *minus* labelled FTIR difference spectra for BlrB and BlrP1-BLUF and their analysis. (**a**) The double difference spectra of the two BLUF domains show a similar pattern of light- and labelling-induced shifts (BlrB: black, BlrP1-BLUF: red). Arrows in the double difference spectrum of BlrB designate the frequency downshifts caused by labelling of at least three difference bands originating either from the dark state (DL) or the light state (LS). (**b**) Fit of the experimental spectrum of BlrB (black) by a sum of six Lorentzians (red). (**c**) Three pairs of the Lorentzians as a result of the fit (black, red, blue). The additional band pair at 1,691(+)/1,672(−) cm^−1^ (red) is not obvious from the experimental spectrum but needs to be included in order to reach a reasonable agreement between the fit and the experiment.

For the light state of BlrB, a single prominent difference band was resolved at 1,697(+)/1,689(−) cm^−1^ in the double difference. However, there is indication for a second prominent difference band of the light state. The observed asymmetry in intensity of the 1,697(+)/1,689(−) cm^−1^ difference band can be explained by a positive contribution of this additional difference band, which overlaps with the 1,689(−) cm^−1^ band and thereby cancels (Fig. 4b). The negative contribution of this additional difference band then adds to the band at 1,667(−) cm^−1^ and explains the broadening observed on the high frequency side of the band. To analyze and verify these additional contributions in the BlrB double difference spectrum, Lorentzians were fitted to the band pattern under the assumption that the oscillator strength is independent of the labelling. Indeed, a match to the experimental results was achievable only by adding an additional band pair (Fig. 4b). The fit yielded band positions of 1,691(+) and 1,672(−) cm^−1^ of this contribution in the deconvolution (Fig. 4c), which indicates a strong shift of the underlying experimental band of the light state of ~20 cm^−1^ by labelling. Such prominent shift is consistent with the characteristic ^15^N shift of the C=N stretch of the imidic acid form of glutamine (Fig. 1). In contrast, a glutamine C=O stretch would only shift a few cm^−1^ in frequency by ^15^N labelling, as we computed for the acetamide molecule (Fig. 2).

### Computational validation of BLUF models

**Figure 5:**
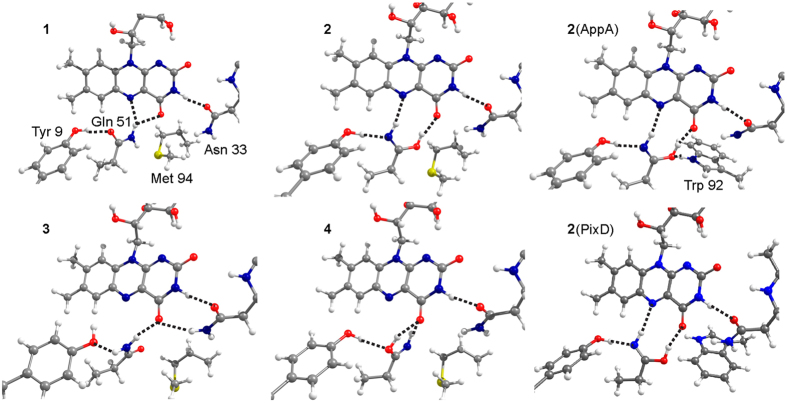
Flavin binding site of BrlB BLUF. Relative orientation of flavin, glutamine amide and Z-Z imidic acid, tyrosine and asparagine in the equilibrium structures. The dashed lines indicate hydrogen bonds, for which the distances are given in Supplementary Fig. S3.

Using the coordinates of the available crystal structure of BlrB in its Met-in conformation^13^, we generated several models (Fig. 5) corresponding to the photoactivation mechanisms discussed in the literature (Fig. 1). We considered the two amide rotamers (models **1** and **3**) and the two Z-Z imidic acid rotamers (models **2** and **4**) of the conserved glutamine in the Met-in active site. Additionally, two Trp-in models were tested with Z-Z imidic acid (models **2**(AppA) and **2**(PixD)) for which the position of the tryptophan side chain was extracted from the crystal structures of the homologous BLUF domains AppA and PixD^10^,^15^. The geometries of the structures were optimised, and the infrared and UV-vis spectra were computed. Model **1**, which reproduces the glutamine orientation proposed in the BlrB crystal structure, has the lowest energy. Moreover, model **1** shows the smallest deviations between the optimised and experimental hydrogen-bonding distances. (The hydrogen bond distances of all models are presented in Supplementary Fig. S3). The energies of models **3**, **2**, and **4** increase relative to the energy of model **1** to 6.1, 8.3, and 13.6 kcal/mol, respectively. The energies of the Trp-in models **2**(AppA) and **2**(PixD) can be compared to each other only. Consequently, we cannot judge whether interactions with tryptophan stabilize the imidic glutamine. Yet, formation of a hydrogen bond between tryptophan and glutamine reduces the energy of model **2**(AppA) by 7.9 kcal/mol in comparison to the energy of model **2**(PixD).

**Figure 6:**
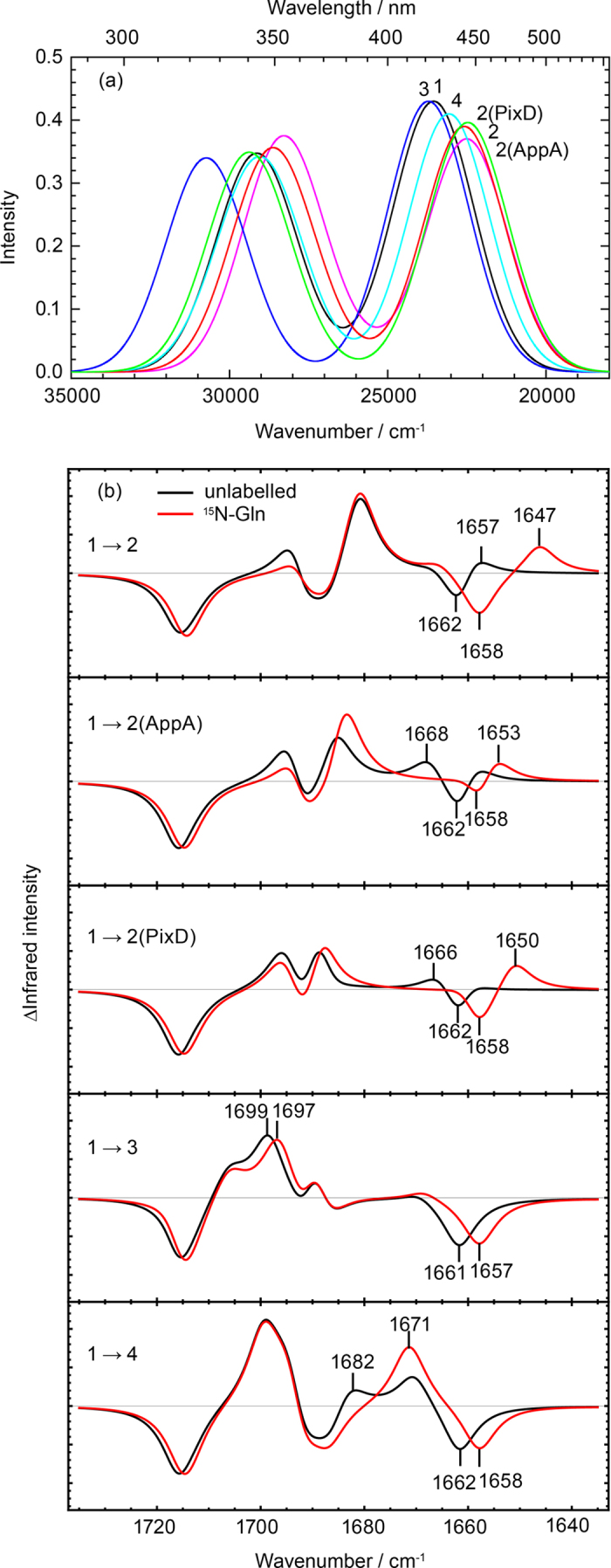
Calculated UV-vis and infrared spectra of flavin binding site models of BlrB BLUF. (**a**) UV-vis absorption spectra computed for the six models shown in Fig. 5. (**b**) Light-*minus*-dark infrared difference spectra with ^15^N-Gln labelling (red) and without labelling (black) for the pairs of models shown in Fig. 5. The strong light-induced downshift of the flavin C_4_=O frequency is reproduced if model **1** with a C_4_=O stretch at 1,716 cm^−1^ is taken as the dark state. The indicated frequency numbers correspond to the C=O and C=N stretches of the glutamine side chain.

We compared the computed S_0_-S_1_ excitation energies and C_4_=O frequencies with the corresponding experimental spectral shifts to assess our models as candidates for the dark and light states. According to our excited state calculations (Fig. 6a), the models that contain the amide glutamine (models **1** and **3**) are characterised by a blue-shifted S_0_-S_1_ absorption maximum, as expected for the dark state. Glutamine tautomerisation leads to a red shift of the S_0_-S_1_ absorption maximum. The most red-shifted maximum, consistent with the experimental 10-nm red shift, is found for the Z-Z imidic acid forming two hydrogen bonds with N_5_ and C_4_=O of flavin (models **2, 2**(AppA) and **2**(PixD)), which, according to this spectral feature, are the best candidates for the light state. For the C_4_=O stretch, the highest frequency was computed for model **1** at 1,716 cm^−1^, in agreement with the experimental frequency in the dark state. All other models demonstrate a significantly downshifted frequency of the C_4_=O stretch. Therefore they would produce in comparison with model **1** a downshifted contribution to the difference spectrum as it is observed in the experiments (Fig. 6b). With respect to model **1**, model **3** demonstrates a 10-cm^−1^ downshift of the C_4_=O stretch, whereas models **2**, **2**(AppA), **2**(PixD) and **4** show a ~20-cm^−1^ downshift.

Importantly, our calculations show that an increase by one unit of a single atomic mass − ^15^N labelling of the glutamine side chain − results in downshifts of several bands (Fig. 6b). To distinguish which of these shifts originate directly from the glutamine stretches and which are caused by coupling through hydrogen bonds, we performed a potential energy distribution analysis (Supplementary Fig. S4). The identified C=O and C=N stretches of glutamine are clearly distinguished by their respective small and large isotopic shifts, whereas their frequencies vary in a rather wide range depending on the hydrogen-bonding interactions. Other ^15^N-induced shifts found in the computed spectra originate from the coupling of the glutamine vibrations with the flavin C_4_=O stretch.

### Computational assignment of the BlrB double difference spectrum

**Figure 7:**
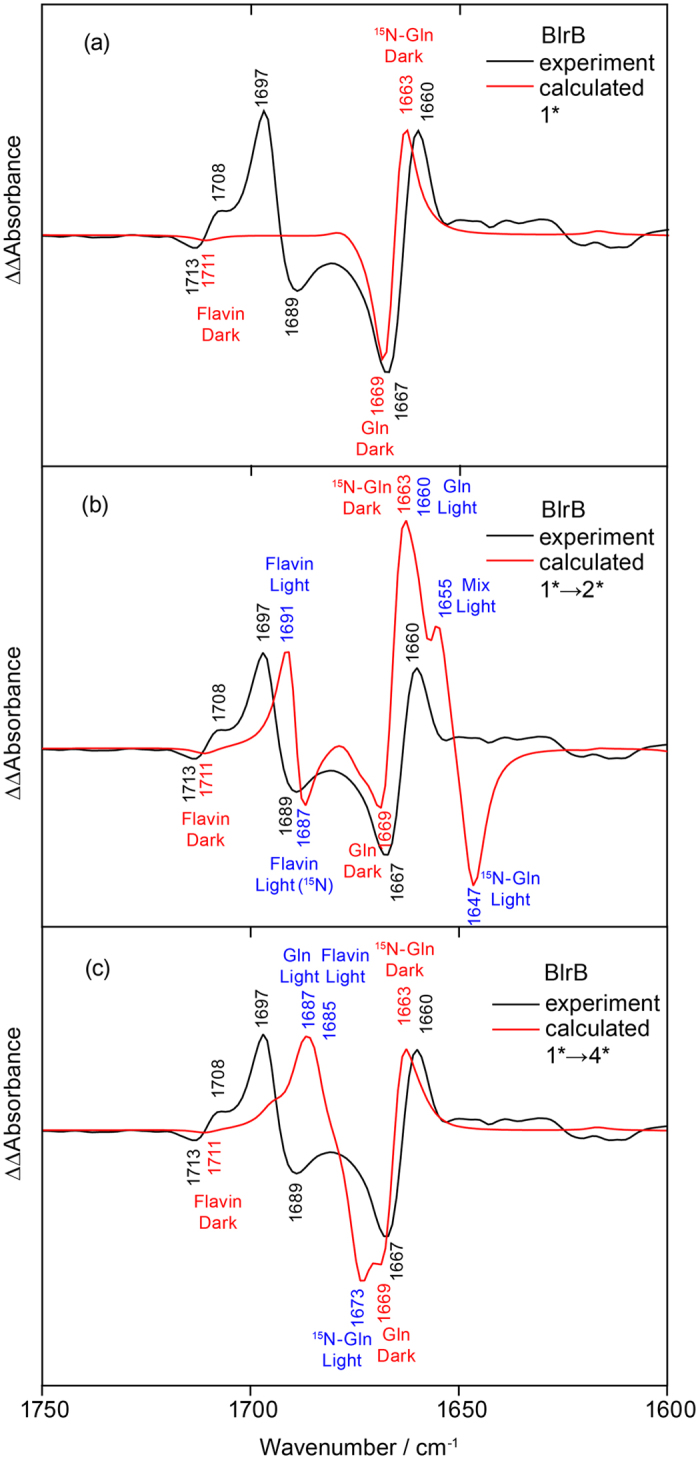
Assignment of the bands shifted by ^15^N labelling in the double difference spectrum of BlrB. Double difference spectra were derived from the calculated vibrational frequencies and infrared intensities by a convolution with Lorentzians with 7 cm^−1^ FWHM (red) for comparison to the experimental result (black). (**a**) Calculations for the effect of ^15^N labelling on the spectrum of model 1* omitting any signals from a conversion to the light state for clarity. (**b**) Calculations for tautomerisation and rotation of glutamine Gln 51 (reaction 1* → 2*). (**c**) Calculations for tautomerisation of glutamine Gln 51 (reaction 1* → 4*). Asterisks indicate extended models including flavin mononucleotide and nine residues (Supplementary Fig. S5).

For the assignment of the experimental double difference spectrum, we focused on the models that reproduced the prominent S_0_-S_1_ and C_4_=O spectral shifts. According to the expected downshift of the C_4_=O frequency, the best candidate for the dark state is model **1**, whereas according to the expected UV-vis red shift, models **2**, **2**(AppA) and **2**(PixD) are plausible candidates for the light state, whilst model **4** demonstrates a somewhat smaller red shift. We created extended active site models of BlrB in the Met-in conformation by including the flavin mononucleotide (FMN) cofactor and nine surrounding residues (Supplementary Fig. S5). The extended models are analogous to the best candidates of the smaller models characterised above, and therefore we label them correspondingly but with an asterisk. Since the Trp-in structure of BlrB or BlrP1-BLUF is not available we did not include the corresponding larger models in our analysis. All key spectral features of the smaller models are reproduced with the larger models (Supplementary Figs S6–S8 and Table S1). With these larger models we obtained the good agreement of the calculated spectra with the experimental double difference spectrum of the BlrB protein (Fig. 7), which eventually enabled us to directly assign all bands to specific vibrations of the glutamine side chain and flavin.

The most prominent band of the dark state at 1,667(−) cm^−1^ is assigned to the Gln 51 C=O stretch computed at 1,669 cm^−1^ (Fig. 7a). The frequency undergoes a moderate downshift by ^15^N labelling in experiment (7 cm^−1^) and theory (6 cm^−1^) because of coupling to the Gln 51 NH_2_ bend. The second, small signal from the dark state at 1,713(−) cm^−1^ results from a weak coupling of flavin C_4_=O computed at 1,711 cm^−1^ with the labelled glutamine. In the extended models this band is rather weak (Fig. 7), but it is more pronounced in the smaller models (Supplementary Fig. S7).

The experimental spectrum of the light state contains a prominent band at 1,697(+) with its labelled counterpart at 1,689(−), therefore exhibiting a small isotope shift. Our calculations suggest an assignment of these bands to the flavin C_4_=O stretch (Fig. 7b). Additionally, the analysis of the experiments revealed a “hidden” band at ~1,691(+) cm^−1^ together with its counterparts at ~1,672(−) corresponding to a large isotopic shift (Fig. 4c). Here, the calculations suggest assignments to the Gln 51 C=N stretch (Fig. 7b). In model 2*, the C=N stretch frequency of the tautomerised and rotated Gln 51 is at 1,660 cm^−1^, which is significantly lower than the frequency extracted from the experiment, whereas the predicted ^15^N downshift of 13 cm^−1^ is in good agreement with the experimentally derived downshift of ~20 cm^−1^. In addition, the calculated flavin C_4_=O frequency at 1,691 cm^−1^ is also lower than the experimental counterpart (Fig. 7a). Model 4* produces the C=N stretch upshifted to 1,687 cm^−1^, which overlaps with the flavin C=O stretches but downshifts by 14 cm^−1^ upon labelling (Fig. 7c). The higher C=N frequency matches the experimentally extracted frequency of ~1,691(+) cm^−1^ much better than the lower frequency derived with model 2*. However, the prominent difference band of the flavin C=O stretch at 1,697(+)/1,689(−) cm^−1^ is lost without Gln 51 rotation. It is conceivable that a further rearrangement of the hydrogen-bonding network is reflected in the experimental spectrum that is not accounted for in our models. For instance, formation of a Trp-in conformation may lead to an upshift of the C=N stretch together with an increase of the ^15^N shift as demonstrated by the comparisons between models **2** and **2**(AppA)/**2**(PixD) (Fig. 6b). However, without having access to a Trp-in structure of BlrB, the effect of the Gln 51−Trp 92 interactions is difficult to evaluate computationally with sufficient accuracy.

## Discussion

### Assignment of the dark state structure of BLUF

Currently, there is no agreement in the community on which glutamine orientation is present in the BLUF dark state. Several previous spectroscopic and biochemical studies favoured a glutamine orientation as suggested by crystal structures of the Trp-in conformation^17^,^19^,^31^. The dark state model postulating this glutamine orientation persists in the literature, because the Trp-in protein conformation of the dark state is in line with the influence of the tryptophan on the decay rate of the flavin excited state^32^. Moreover, Raman and fluorescence spectroscopic experiments indicated that tryptophan is positioned in a buried environment in the dark^33^,^34^, but with conflicting results on whether this changes in the light state^33^,^34^,^35^. For the structure of the hydrogen-bonding network, the Trp-in orientation^10^ implies that glutamine NH_2_ donates a hydrogen bond to tyrosine. However, this Trp-in orientation of glutamine in the dark state is challenged by the fact that the Met-in protein conformation is more commonly found in the crystal structures. There, the glutamine side chain is proposed to accept a hydrogen bond from the tyrosine O−H group. Indeed, BlrB and BlrP1-BLUF studied here were both observed to adopt the Met-in conformation in the dark in the crystal and in solution^11^,^13^,^36^,^37^. The Met-in orientation of the glutamine side chain is stable upon quantum-chemical geometry optimization in contrast to the Trp-in orientation^38^.

The structure of the flavin binding pocket of BLUF is encoded in the light-induced infrared difference spectrum, in which the characteristic downshift of the prominent flavin C_4_=O stretch has been in the focus of the attention. This band and others have been assigned in the meantime by uniformly labelling the protein and by specifically labelling the flavin or the conserved tyrosine^9^,^39^,^40^,^41^,^42^,^43^ as well as by quantum chemical calculations^28^,^39^,^44^. However, the infrared signature of the conserved glutamine could not be unambiguously identified yet. A band at 1,666(−) cm^−1^ previously observed by ultrafast infrared spectroscopy in AppA-BLUF was tentatively assigned to the conserved glutamine because of its absence in the photoinactive Gln 63 Leu mutant as well as in the light state of AppA-BLUF^19^.

**Figure 8:**
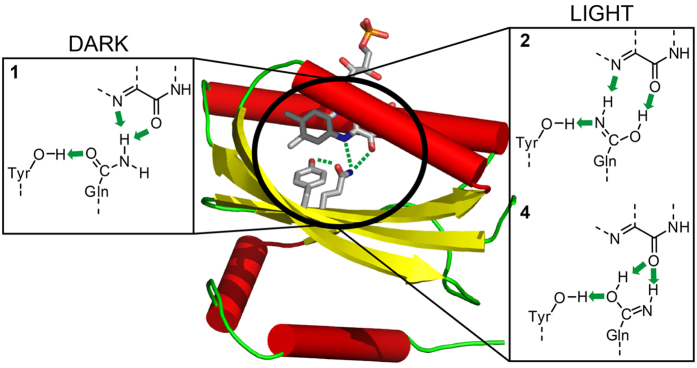
The dark and light state structures of the flavin binding pocket in BLUF as derived from vibrational spectroscopy and quantum chemical calculations. The BLUF domain of BlrB is depicted with the flavin binding site (PDB ID 2BYC). The close-ups show the dark state structure 1 and the light state structures 2 and 4 with the Z-Z imidic acid form of glutamine stabilised by hydrogen-bonding interactions (green arrows).

In the BlrB double difference spectrum resulting from photoactivation and labelling, we identified the glutamine band at 1,667(−) cm^−1^ in the dark state. Such a frequency is low for a glutamine, similar to the 1,670 cm^−1^ resulting from hydrogen bonds in water^45^. The congruence between experiment and theory on the frequency of the glutamine C=O stretch at 1,667 cm^−1^ presented here provides direct evidence for a BLUF configuration as in model **1** in which the glutamine C=O points to the tyrosine and accepts a hydrogen bond (Fig. 8). The presence of such a hydrogen bond configuration in the dark state is also in line with previous, systematic infrared spectroscopic studies on the tyrosine vibrations in BLUF^43^. Moreover, model **1** reproduces the rather high flavin C_4_=O frequency and its coupling to the glutamine vibrations observed as a weak, second contribution to the double difference spectrum at 1,713 cm^−1^. Finally, model **1** exhibits the blue-shifted flavin S_0_-S_1_ absorption maximum. Thus, infrared spectroscopy combined with ^15^N labelling and quantum chemical calculations provides evidence for the Met-in orientation of the conserved glutamine in the dark state.

### Assignment of the light state structure of BLUF

Assignment of the glutamine orientation of model **1** to the dark state rules out glutamine rotation as the photoreaction mechanism. From all the mechanisms proposed in the literature (Fig. 1), only reactions featuring a formation of the imidic acid glutamine tautomer remain. The respective molecular models **2** and **4** featuring the Z-Z imidic acid reproduce the light-induced red shift in the UV-vis spectrum and the downshift of the flavin C_4_=O stretch.

The infrared signature of glutamine in the light state is identified in this study, to our knowledge, for the first time. Therefore, we cannot compare our glutamine assignment with those of others. We identified that in the light state, the glutamine absorbs at ~1,691(+) cm^−1^. The large ^15^N shift of the latter band to ~1,672(−) cm^−1^ argues for a photochemical modification of the glutamine to its imidic acid tautomeric form (Fig. 8). The presence of these positive and negative signals in the double difference spectrum is derived from the asymmetry of the negative bands at 1,689 and 1,667 cm^−1^ with respect to their positive counterparts at 1,697 and 1,660 cm^−1^.

The additional higher frequency pair at 1,697(+)/1,689(−) cm^−1^ in the double difference spectrum was explained by the coupling between the C=N and flavin C_4_=O stretches. Hence, the flavin C_4_=O frequency is downshifted to 1,697 cm^−1^ in the light state by a hydrogen bond with the imidic acid glutamine. The rather significant downshift of this band by ^15^N labelling of glutamine provides evidence for enhanced hydrogen-bonding interactions between flavin and the protein moiety in the light state. However, our assignment of the signal at 1,697(+) cm^−1^ to flavin C_4_=O is in conflict with previous assignments that locate this stretch at 1,684 cm^−1^ in the light state of AppA-BLUF^30^. The latter signal was identified by global labelling the protein moiety with ^13^C but keeping the flavin at natural isotope abundance to shift overlapping contributions of the protein amide modes. We have computationally performed the same mass substitutions and found a downshift of the vibrational frequency of the flavin C_4_=O in the light state model **2** (but not in model **4**) despite flavin remaining at natural abundance (Supplementary Fig. S9), thus reconciling the different assignments. As an alternative to be assessed, the 1,697(+) cm^−1^ band could be assigned to the glutamine C=O stretch in the light state. Such a frequency is exceptionally high for a glutamine. Yet, the experiments on the homologous BlrP1-BLUF point to an even higher band position at 1,705 cm^−1^, which makes its assignment to glutamine implausible. Moreover, the strong asymmetry in the intensities of the double difference bands would require some credible explanation.

Finally, Iwata *et al.*^42^ demonstrated the exceptionally low frequency of the tyrosine O−H stretch at 2,800–2,600 cm^−1^ in the light state, whereas a typical position would be at 3,600–3,200 cm^−1 ^42. Such a low frequency points to an unusually strong hydrogen bond formed between tyrosine and most likely glutamine. In our calculations, such a lowering in the frequency is indeed found exclusively when tyrosine donates a hydrogen bond to the C=N imidic moiety of the tautomerised and rotated glutamine (model **2**) (Supplementary Table S2).

### Reappraisal of the approach combining isotopic labelling, infrared spectroscopy and quantum chemical calculations

The general approach to analyze the frequency shifts induced by isotope labelling in the infrared spectrum is a powerful tool known as isotope-edited infrared spectroscopy and has provided valuable insight into enzyme mechanisms beyond photochemical processes^46^,^47^. Nonetheless, the interpretation of the resulting infrared double difference spectrum is not always straightforward as it is demonstrated in our study. Complications originate from the coupling of the labelled chemical group to its molecular environment. At first approximation one might consider only the covalent bonds to the labelled atom, thereby gaining the possibility to, e.g., effectively separate contributions from cofactor and protein moiety^30^. In our case, only two difference bands would then have been expected by ^15^N-Gln labelling instead of the four band pairs detected. The additional signals might erroneously be attributed to a scrambling of the isotopic label during protein biosynthesis. Yet, our extensive quantum chemical calculations provide the assignment of these additional signals to a functional group within the hydrogen-bonding network of the labelled compound without invoking label scrambling. It should be noted that the coupling in the hydrogen-bonding network is rather counterintuitive as it does not necessarily provide a measure of the distance between the isotopic label and the coupled functional group. For instance, our analysis revealed that the coupling of glutamine ^15^N is stronger to flavin C_4_=O in model **2** than in model **4**, despite the fact that in the latter geometry these groups directly form a hydrogen bond (Fig. 8). Therefore, additional information is provided by the spreading of the isotope-induced shifts, which is crucial to identify the molecular structure and the strength of interaction in the hydrogen-bonding network. Such information is particularly valuable in cases such as the photoreaction activating the BLUF domain, where a strong and specific chemical coupling between the cofactor and the protein by means of glutamine tautomerisation provides a basis for light sensitivity.

## Conclusions

We identified the vibrational signatures of the conserved glutamine in the dark and light state by performing ^15^N labelling of all glutamines in two representative BLUF proteins. Combination of infrared spectroscopic experiments with quantum chemical calculations of different proposed BLUF active site models enabled us to conclude that photoactivation is achieved via tautomerisation of the conserved glutamine. In the light state, two ^15^N-sensitive frequencies at 1,697 and ~1,691 cm^−1^ are assigned to the flavin C_4_=O and glutamine C=N stretches, respectively. This assignment settles a long-lasting debate about the hydrogen-bonding structure of the dark and light states of the BLUF domain.

BLUF photoactivation might be regarded as a conversion of the energy of the photon into an increase of the number of hydrogen bonds in the flavin binding pocket. An additional stabilising mechanism might involve a proton transfer event in the protein moiety outside the flavin-binding pocket occurring on a sub-millisecond timescale as suggested previously^48^. It remains to be clarified how the metastable imidic acid tautomer with its specific hydrogen bonds triggers the conformational switch and becomes sufficiently stabilised to sustain a long-lived signalling state that is capable of initiating a variety of physiological responses.

## Methods

### Protein expression and purification

A strain of *E. coli* BL21(DE3) auxotrophic for glutamine was prepared as previously described^49^ using the *glnA* knockout gene from *E. coli* strain JW3841 (obtained from CGSC)^50^. The resulting cells carrying the *glnA* knockout allele were electroporated with an expression plasmid that contained the full-length BlrB gene (coding for amino acids 1–140)^51^ or the BlrP1-BLUF gene (coding for amino acids 1–137)^52^ cloned into the pET28a(+) plasmid (Novagen). For protein expression, cells were grown in 5 L of minimal medium supplemented with 150 mg/L L-glutamine-amide-^15^N (98% ^15^N, Aldrich) to an OD_600_ of 0.16, at which time isopropyl-β-d-thio-galactopyranoside was added to 0.5 mM. The rest of the protein isolation and purification followed the protocol described previously^51^, with the following minor variations. The protein as eluted from the affinity column was dialyzed against 25 mM Tris-HCl, pH 7.5, 75 mM NaCl, and 5 mM MgCl_2_. Additionally, flavin adenine dinucleotide was added to 100 mM for full reconstitution, and 300 U thrombin (Sigma) was added for the removal of the His-tag. Tag-free protein was concentrated and purified on a Superdex 75 HR 16/60 column (GE Life Sciences) in 25 mM Hepes pH 7.5, 100 mM NaCl, 5 mM MgCl_2_, 2.5 mM ethylenediaminetetraacetic acid, 2 mM dithioerythritol and 10% glycerol. Finally, the protein was dialyzed against 15 mM sodium phosphate, pH 8.0, 10 mM NaCl and stored at −80 °C at a concentration of 30 mg/mL for BlrB and 15 mg/mL for BlrP1-BLUF. The degree of incorporation of ^15^N in glutamine was investigated by mass spectrometry (MALDI-TOF intact mass and peptide map fingerprinting) and found to be 75% for BlrB and 58% for BlrP1-BLUF. Some scrambling of the label to other amino acids, especially asparagine, might have occurred during expression lowering these yields. However, the infrared difference experiments and the following analysis by quantum chemical calculations explicitly including this scrambling did not provide any evidence for such contributions (Supplementary Fig. S10). Unlabelled BlrB and BlrP1-BLUF (i.e., at natural isotope abundance) were obtained following the same procedure.

### Rapid-scan time-resolved Fourier transform infrared (FTIR) spectroscopy

The concentrated protein solutions, 3 μL of BrB or 4 μl of BlrP1-BLUF, were applied to a BaF_2_ disc and were gently reduced in water content at a pressure of 500 mbar for 5 min for unlabelled BlrB, for 8 min for ^15^N-Gln BlrB, and for 12 min for unlabelled and ^15^N-Gln BlrP1-BLUF. This procedure preserves the protein in its solution environment with high water content (Supplementary Fig. S11). A sandwich cuvette was obtained by sealing with a second BaF_2_ disc leading to a pathlength of ca. 10 μm. The absorbance at 1,645 cm^−1^ was adjusted to 0.8–0.9 for all samples.

FTIR spectra were recorded with a Bruker IFS 66s spectrometer equipped with an MCT detector at a scanning velocity of 320 kHz and a resolution of 2 cm^−1^. A long wave pass filter was placed in front of the detector to block stray light and to improve the signal-to-noise ratio by restricting the recording range to <2,000 cm^−1^. The samples were illuminated with an intensity of 20 mW/cm^2^ for 1 s by a 455-nm LED for BlrB and 445-nm LED for BlrP1-BLUF with a homogenised light profile by passage through a scattering disc. Temperature was maintained at 20 °C by a circulating water bath. Before and after illumination, a series of rapid-scan spectra were recorded with 12 scans each for BlrB and 256 scans each for BlrP1-BLUF until the signals of the samples had decayed completely. Averaged difference spectra were calculated from the first scans directly after *minus* those before illumination corresponding to time intervals after illumination of 0.01–1.35 s for BlrB (12 scans) and 1.36–30.21 s for BlrP1-BLUF (256 scans). The sample was kept in the dark for 25 s for BlrB and 261 s for BlrP1 to avoid excitation of the photoproduct in the next measurement cycle. In total, representative spectra were produced from 2,160 averaged scans for unlabelled and ^15^N-Gln BlrB, 35,328 scans for unlabelled BlrP1, and 42,240 scans for ^15^N-Gln BlrP1-BLUF, recorded each on three independent samples. The different protocol for the two samples is a consequence of the differences in the photocycle kinetics. Later spectra in the time sequence showed a very similar pattern to that of the first time interval pointing to a homogenous decay (Supplementary Fig. S2).

### Fit to the Experimental Double Difference Spectrum

A sum of six Lorentzians was fit to the experimental data in the range of 1,650–1,700 cm^−1^ using the function


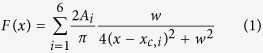


in OriginPro 7.5 (OriginLab). The peak positions *x*_c_ of the four experimentally determined bands were kept fixed during the fitting process as well as the area *A* of the band at 1,697 cm^−1^. The ratio of the area of the positive band to that of the negative band within a band pair was kept at unity. Five free parameters were varied including *w*, the full width at half maximum (FWHM) of all Lorentzians, which resulted in *w* = 7 cm^−1^.

### Quantum chemical calculations

To aid in the spectral assignment, geometry optimization and vibrational infrared spectra calculations in the harmonic approximation were performed for a number of molecular models using the B3LYP/6-31G(d,p) method. The Firefly quantum chemistry package version 8.1^53^, which is partially based on the US-GAMESS^54^ program source code, was used in all calculations.

To compare the shifts induced by ^15^N labelling in the amide and Z-Z imidic acid, we computed the respective isomers of acetamide. Two model systems were considered to include the solvent and hydrogen-bonding effects: the acetamide or its imidic acid embedded in dielectric PCM water^55^, and acetamide or its imidic acid in a complex with two D_2_O molecules embedded in PCM water. In the latter case, acetamide and imidic acid were considered in a hydrogenated form, whereas water molecules were considered in a deuterated form (Fig. 2c,d) to downshift water bending frequencies otherwise overlapping with the amide/imidic acid stretching and bending frequencies.

Further, we prepared two series of supermolecular cluster models of the BlrB BLUF domain. The smaller cluster models include the riboflavin (RF) chromophore together with its essential environment common to all BLUF proteins: side chains of Tyr 9, Gln 51, Met 94 (or Trp 92), Ser 29, Ile 25, and Asn 33. In addition, we computed three cluster models that were larger in size, containing the flavin mononucleotide (FMN) chromophore instead of RF and Arg 32, Leu 63 and Ser 93 in addition to the side chains specified above. The starting geometries of all models were prepared using the same PDB coordinates of the X-ray crystal structure coordinates of the BlrB protein in its Met-in conformation (PDB ID 2BYC, molecule A). To model Gln 51 modifications, the PDB coordinates of the oxygen and nitrogen atoms were exchanged and the coordinates of the hydrogen atoms were generated according to a specific tautomeric form using the model-build tool of the HyperChem program version 7.52 (Hypercube Inc. 2002). To model the Trp-in binding pocket, the AppA^10^ (PDB ID 1YRX, molecule A) and PixD BLUF^15^ (PDB ID 2HFN, molecule D) structures were used as templates. The starting coordinates of the Trp 92 side chain in models **2**(AppA) and **2**(PixD) were obtained by superimposing the BlrB cluster model with the respective Trp-in crystal structures. During geometry optimization, the coordinates of several terminal atoms (Supplementary Fig. S5) were kept as in the PDB structure in order to maintain the geometry as close as possible to the starting geometry. At the optimised geometries, the harmonic vibrational analysis was performed.

For comparison with the experimental double difference spectra, ^15^N labelling of the Gln 51 side chain was performed. The computed frequencies were scaled by 0.965. The infrared spectra were obtained by convolution with Lorentzian functions of 8 cm^−1^ FWHM. The normal modes were assigned to the vibrations of specific functional groups by normal mode decomposition and potential-energy distribution analysis^56^, which were carried out using an identical set of intrinsic coordinates (bonds, angles and torsions) for all cluster models generated with an automated procedure (dlc = .true.)^57^.

At the equilibrium geometries of the BlrB models, the excitation energies and oscillator strengths of the electronic transitions were computed with the XMCQDPT2 method^58^ using a previously established protocol^59^,^60^. Four low-energy electronic states were included in the calculations with the CASSCF(6,4)4 zeroth-order wave function, i.e., the closed-shell ground state, the two locally excited states of flavin (the HOMO-LUMO and (HOMO-1)-LUMO transitions) and the intermolecular electron transfer state from tyrosine to flavin (the HOMO(Tyr 9) to LUMO(flavin) transition). For the models containing Trp 92, five states were computed with the CASSCF(8,5)5 zeroth-order wave function by adding to the four states specified above another electron transfer state from tryptophan to flavin (the HOMO(Trp 92) to LUMO(flavin) transition). From the results of the excited-state calculations, the UV-vis spectra were simulated using a convolution with Gaussian functions of 3,000 cm^−1^ FWHM.

## Additional Information

**How to cite this article**: Domratcheva, T. *et al.* Evidence for Tautomerisation of Glutamine in BLUF Blue Light Receptors by Vibrational Spectroscopy and Computational Chemistry. *Sci. Rep.* **6**, 22669; doi: 10.1038/srep22669 (2016).

## Supplementary Material

Supplementary Information
