# Supplementary material for: Evidence for Tautomerisation of Glutamine in BLUF Blue Light Receptors by Vibrational Spectroscopy and Computational Chemistry
Source: Sci Rep. 2016 Mar 7;6:22669. doi: 10.1038/srep22669 (PMC4780082; doi:10.1038/srep22669)
Supplement: Supplementary Information [file srep22669-s1.pdf]

Supplementary Information

Evidence for Tautomerisation of Glutamine in BLUF Blue Light Receptors by Vibrational Spectroscopy and Computational Chemistry

Tatiana Domratcheva[1,*], Elisabeth Hartmann[1], Ilme Schlichting[1], and Tilman Kottke[2,*]

[1]Department of Biomolecular Mechanisms, Max Planck Institute for Medical Research, Jahnstrasse 29, 69120, Heidelberg, Germany.

[2]Physical and Biophysical Chemistry, Department of Chemistry, Bielefeld University, Universitätsstraße 25, 33615 Bielefeld, Germany.

*Corresponding authors: Tatjana.Domratcheva@mpimf-heidelberg.mpg.de, Tilman.Kottke@uni-bielefeld.de.

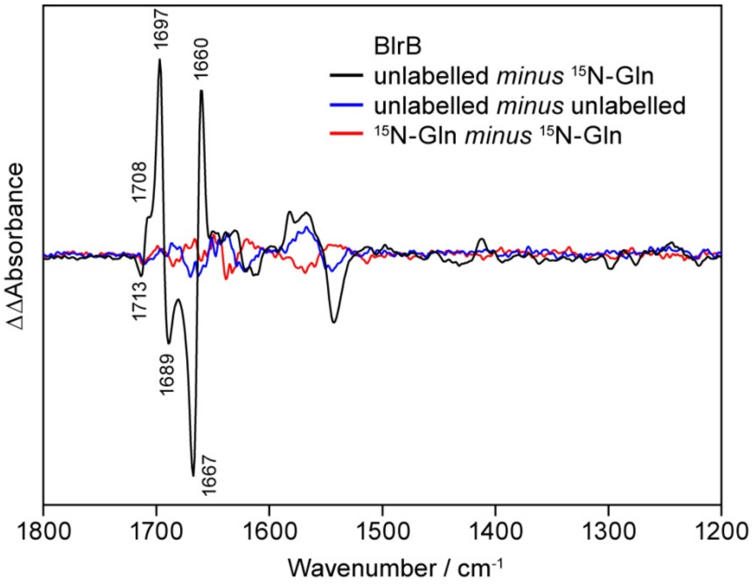

Supplementary Figure S1. Sample to sample variation of experimental double difference spectra. Difference spectra from independent preparations were scaled by an unbiased least squares fit (1,500-1,200 cm^{-1}) and subtracted from each other as indicated. All signals originating from ^{15}N-Gln labelling at > 1,600 cm^{-1} are far above the variation from sample to sample.

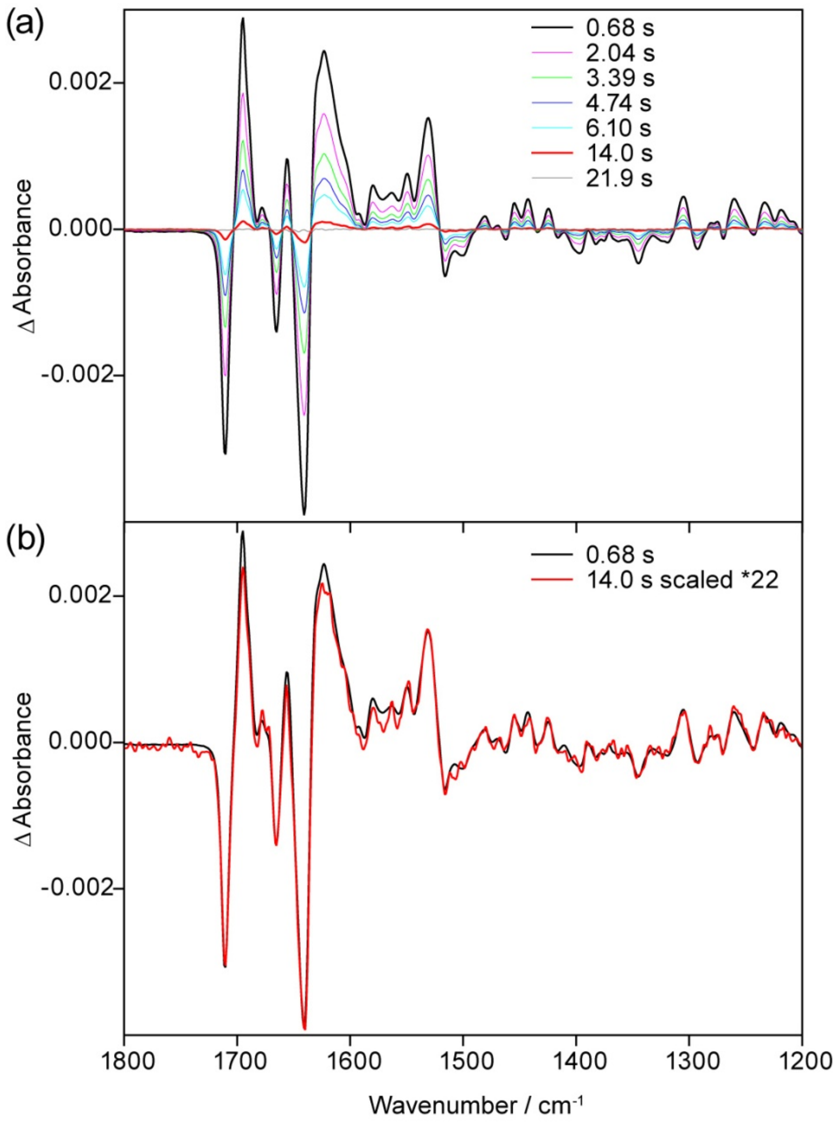

Supplementary Figure S2. Representative, time-dependent FTIR difference spectra of BlrB after illumination. (a) The spectrum at 0.68 s was taken for analyzing the effects of ^{15}N-Gln labelling. The light state completely decayed within 22 s. (b) A comparison of early and late spectra in the decay of the light state reveals a slightly faster decay of bands in the amide I and amide II region than those <1,500 cm^{-1}. Therefore, changes in secondary structure of BlrB relax only slightly faster than those taking place at the flavin. Any indications for further structural rearrangements of the light state are not evident during the homogeneous decay.

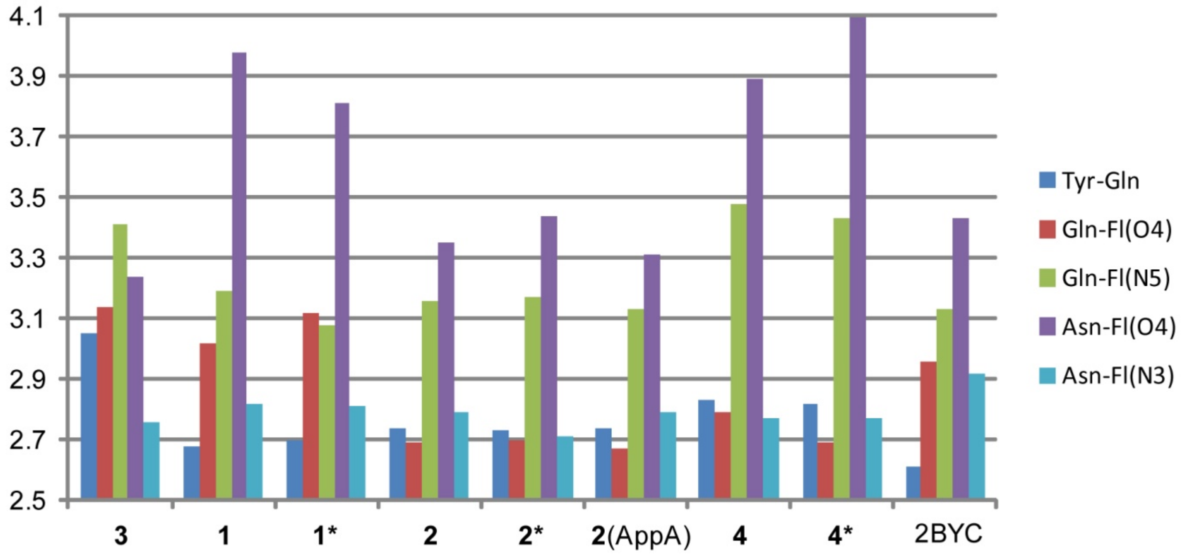

Supplementary Figure S3. Interatomic distances (Å) between the electronegative atoms of hydrogen bonds around flavin in the smaller BLUF models, the extended BLUF models (indicated by an asterisk) and in the BlrB crystal structure (PDB ID: 2BYC, molecule A).

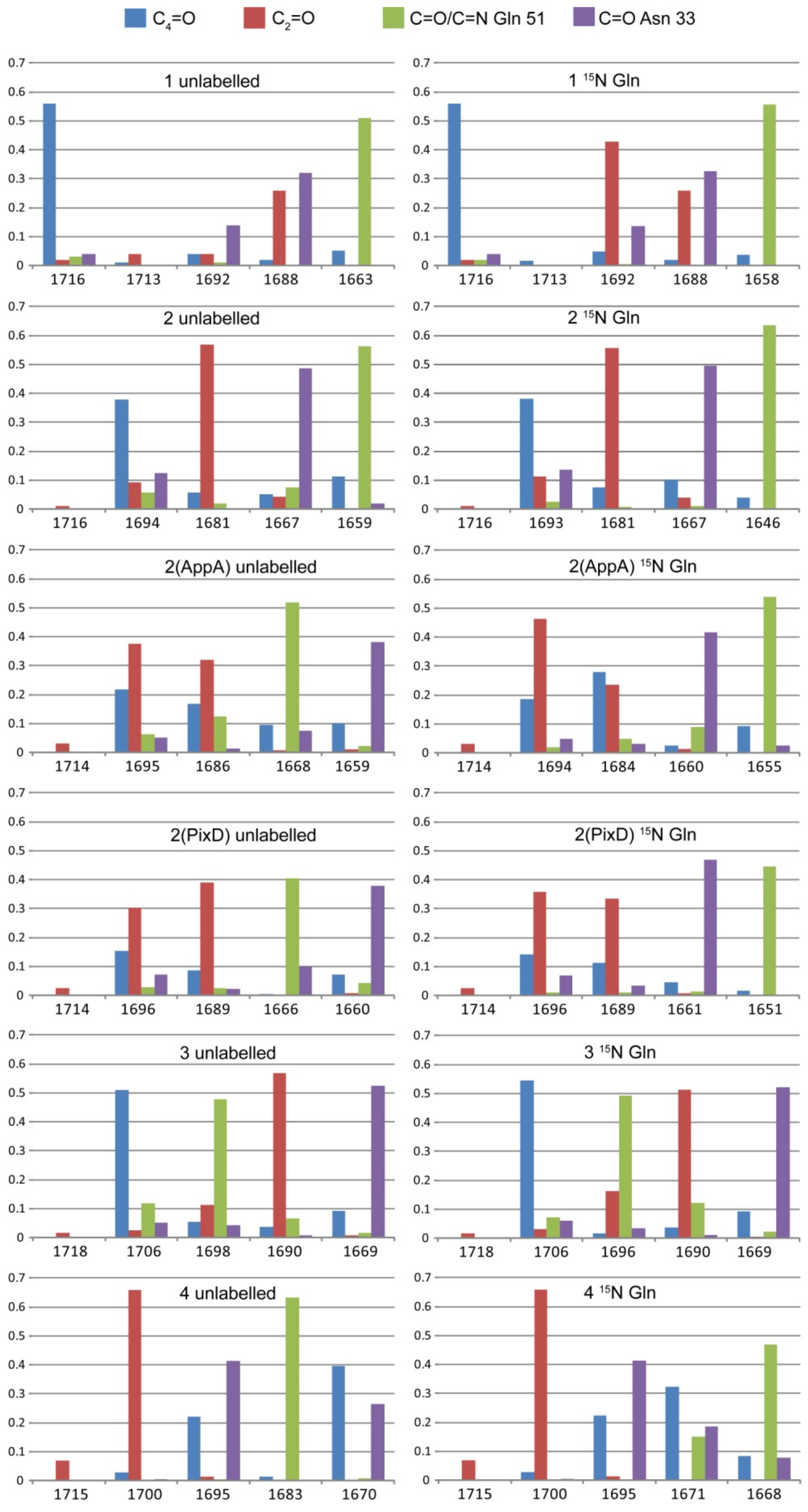

Supplementary Figure S4. Potential energy distribution analysis for models **1**, **2**, **2**(AppA), **2**(PixD), **3**, and **4**. Frequencies (cm^{-1}) are scaled by 0.965.

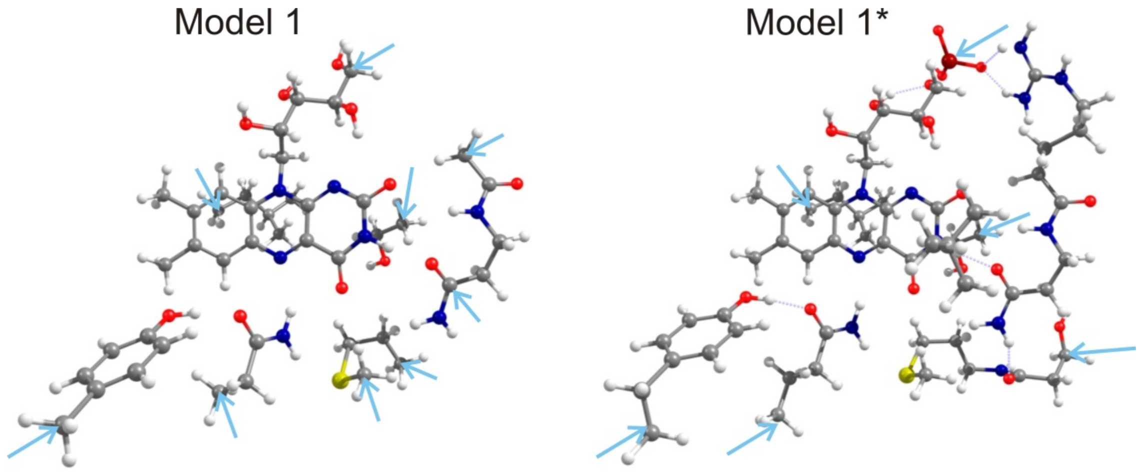

Supplementary Figure S5. Supramolecular cluster models of the BlrB BLUF domain as shown exemplarily for model **1**. The smaller cluster (left) comprised riboflavin and side chains of Tyr 9, Gln 51, Met 94, Ser 29, Ile 25, and Asn 33. The extended model **1*** (right) contained flavin mononucleotide (FMN) and Arg 32, Leu 63 and Ser 93 in addition to the side chains specified above. The coordinate locking employed in the geometry optimization is shown. The blue arrows point at the atoms with Cartesian coordinates frozen in geometry optimization. Identical constraints were applied in all computed models.

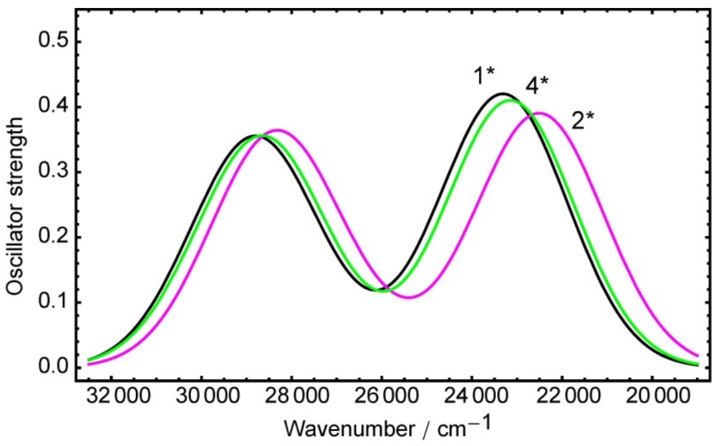

Supplementary Figure S6. Computed absorption spectra for the extended models of the BlrB active site.

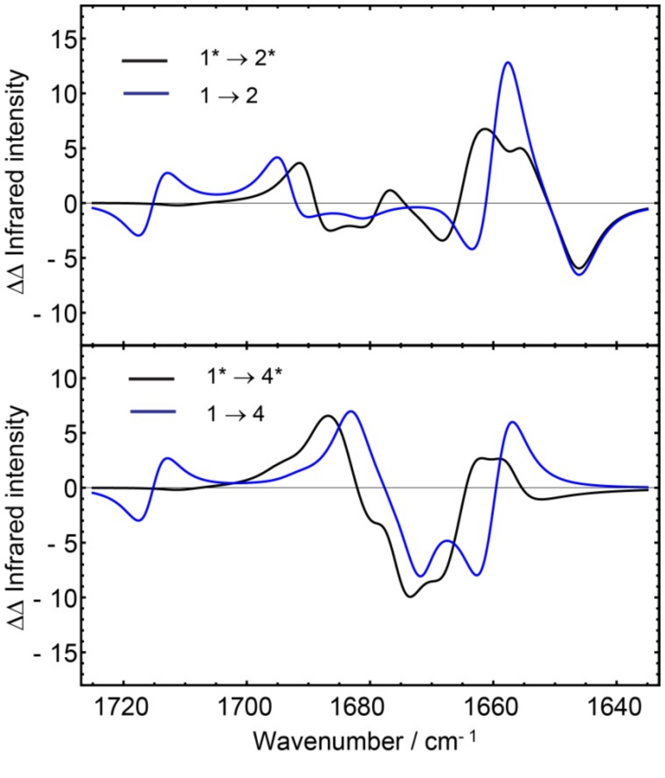

Supplementary Figure S7. Comparison of the ^{15}N-Gln shifts in the smaller BLUF models (blue) and the extended models (black, indicated by an asterisk). Double difference spectra (unlabelled *minus* ^{15}N-Gln) calculated from the respective light *minus* dark difference spectra.

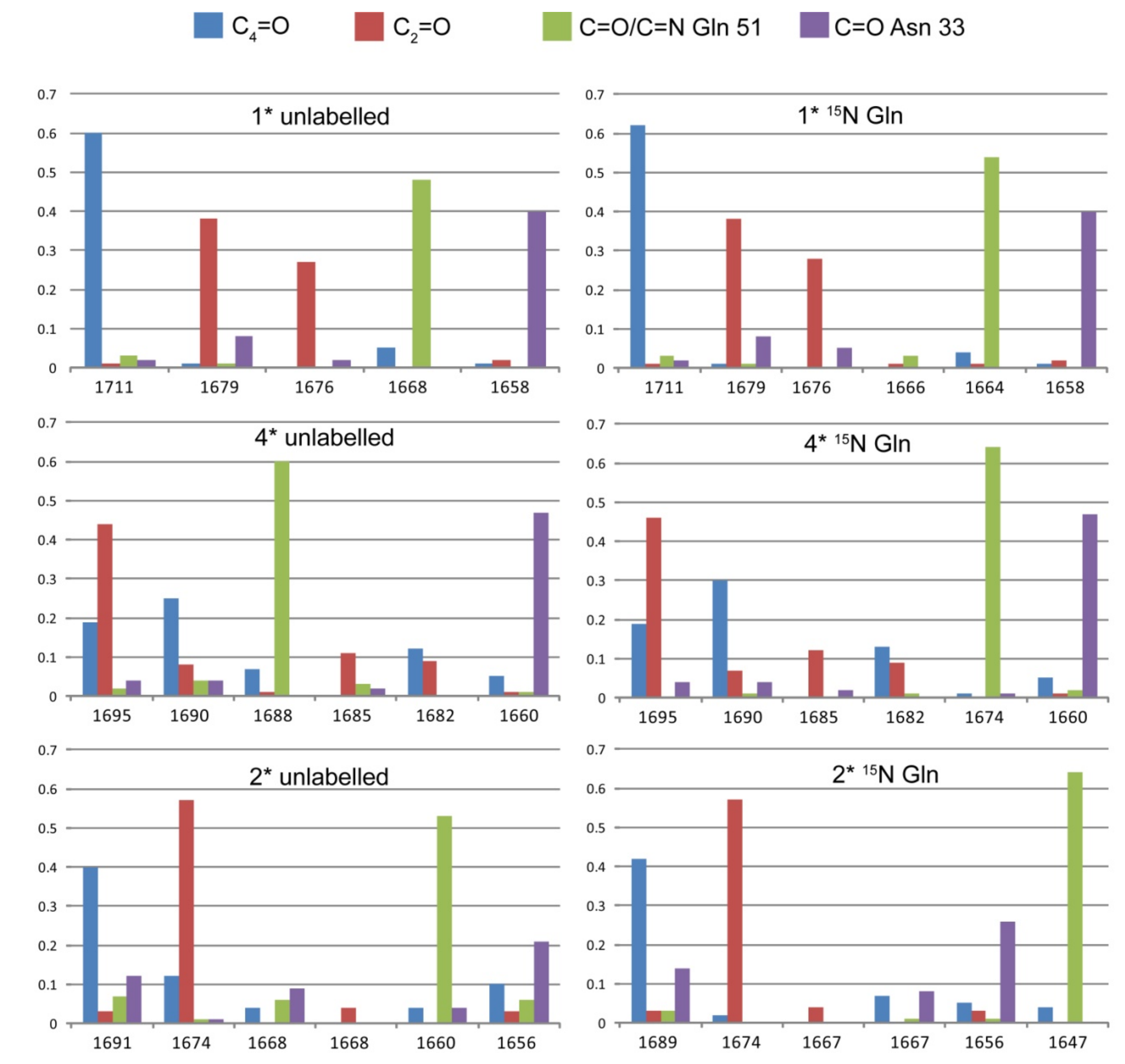

Supplementary Figure S8. Potential energy distribution analysis for the extended models **1***, **2*** and **4***. Frequencies (cm^{-1}) are scaled by 0.965.

Supplementary Table S1. Potential energy distribution analysis for the extended models **1***, **2*** and **4*** (same data as in Fig. S8). Frequencies are scaled by 0.965. Prominent contributions to the calculated double difference spectra presented in Fig. 7 are highlighted.

Model	Normal mode frq. cm^{-1}	Intrinsic coordinates			
		C_4=O_4	C_2=O_2	C=O/C=N(Gln)	C=O(Asn)
**1*** unlabelled	1711	**0.60**	0.01	0.03	0.02
	1679	0.01	0.38	0.01	0.08
	1676	0.00	0.27	0.00	0.02
	1668	0.05	0.00	**0.48**	0.00
	1658	0.01	0.02	0.00	0.40
**2*** unlabelled	1691	**0.40**	0.03	0.07	0.12
	1674	0.12	0.57	0.01	0.01
	1668	0.04	0.00	0.06	0.09
	1668	0.00	0.04	0.00	0.00
	1660	0.04	0.00	**0.53**	0.04
	1656	0.10	0.03	0.06	0.21
**4*** unlabelled	1695	0.19	0.44	0.02	0.04
	1690	0.25	0.08	0.04	0.04
	1688	0.07	0.01	**0.60**	0.00
	1685	0.00	0.11	0.03	0.02
	1682	**0.12**	0.09	0.00	0.00
	1660	0.05	0.01	0.01	0.47
**1*** ^{15}N-Gln	1711	**0.62**	0.01	0.03	0.02
	1679	0.01	0.38	0.01	0.08
	1676	0.00	0.28	0.00	0.05
	1666	0.00	0.01	0.03	0.00
	1664	0.04	0.01	**0.54**	0.00
	1658	0.01	0.02	0.00	0.40
**2*** ^{15}N-Gln	1689	**0.42**	0.03	0.03	0.14
	1674	0.02	0.57	0.00	0.00
	1667	0.00	0.04	0.00	0.00
	1667	0.07	0.00	0.01	0.08
	1656	0.05	0.03	0.01	0.26
	1647	0.04	0.00	**0.64**	0.00
**4*** ^{15}N-Gln	1695	0.19	0.46	0.00	0.04
	1690	0.30	0.07	0.01	0.04
	1685	0.00	0.12	0.00	0.02
	1682	0.13	0.09	0.01	0.00
	1674	0.01	0.00	**0.64**	0.01
	1660	0.05	0.01	0.02	0.47

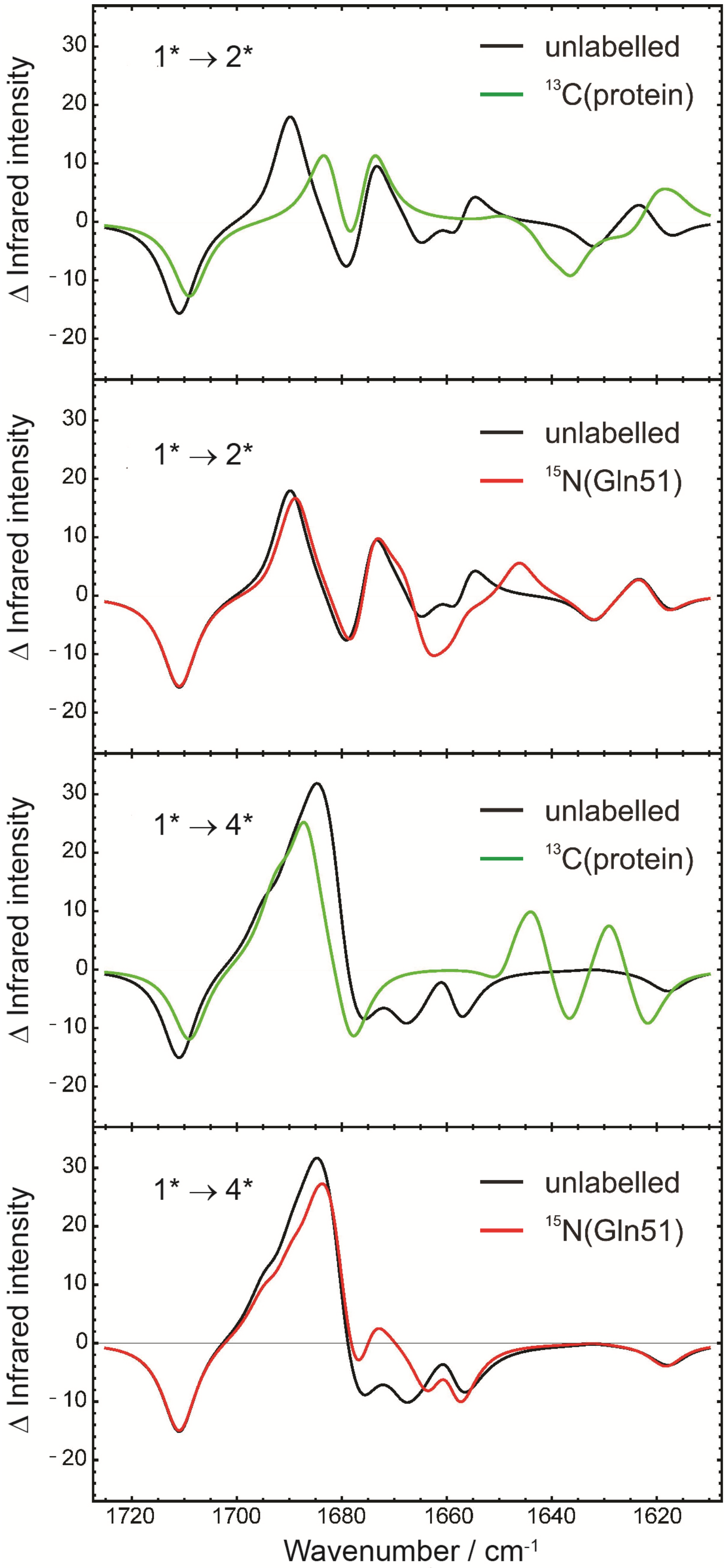

Supplementary Figure S9. Simulated light-*minus*-dark infrared difference spectra of unlabelled, ^{15}N(Gln) and ^{13}C(protein) labelled BLUF. Two photoactivation reactions are considered − glutamine tautomerisation (**1*→4***) and glutamine tautomerisation and rotation (**1*→2***). The flavin C_4=O stretch at 1711(-) cm^{-1} displays a downshift upon ^{13}C labelling of the protein despite the fact that the flavin remained unlabelled,

Supplementary Table S2. Computed frequency of the Tyr 9 OH stretch

Model	Frequencies (cm^{-1}) scaled by 0.965
**1**	3263
**2**	3010
**2**(AppA)	3052, 3054, 3059 (mixed with Tyr9 CH stretches)
**2**(PixD)	3007
**3**	3680
**4**	3473
**1***	3342, 3313 (mixed with Gln51 NH_2 sym. stretch)
**2***	3012
**4***	3455

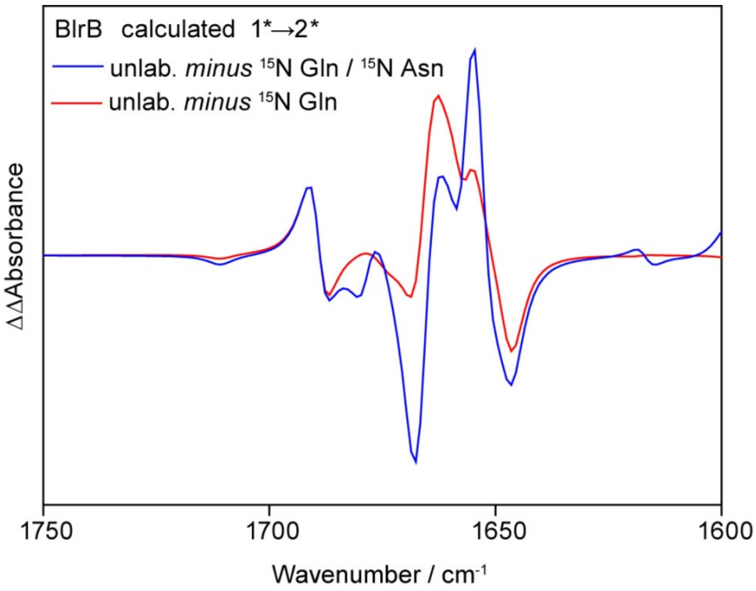

Supplementary Figure S10. Double difference spectra (unlabelled *minus* ^{15}N-Gln/^{15}N-Asn, blue) calculated from the respective light *minus* dark difference spectrum for the reaction of model **1*** to model **2***. Hypothetical full scrambling of the ^{15}N label from Gln to Asn might lead to additional ^{15}N labelling of Asn33. The comparison to the double difference considering only ^{15}N labelling of Gln (black) demonstrates that such scrambling would lead to a more complex signal pattern, which is not observed in the experiment. The weak shift of Asn33-CO frequency by labelling cannot account for the large shift in this spectral region as extracted from the experiment (Fig. 4). Furthermore, the experimental band at 1697(+) / 1689(-) cm^{-1} does not contain contributions by Asn33, which supports the assignment to flavin-CO frequencies shifted by indirect coupling to ^{15}N Gln.

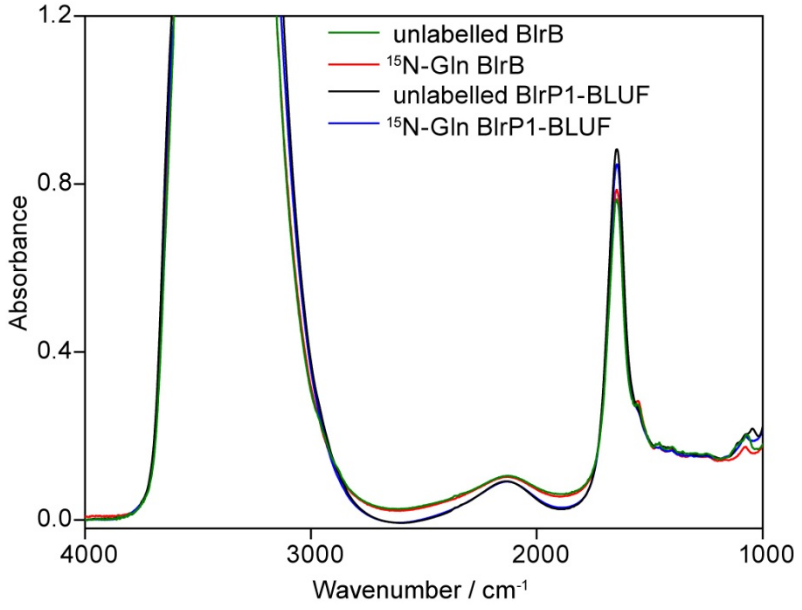

Supplementary Figure S11. Representative infrared absorption spectra of BlrB and BlrP1-BLUF. Protein samples were investigated well-hydrated in solution as is evidenced by the very high absorbance ratio of water / amide I (1,650 cm^{-1}) to amide II (1,550 cm^{-1}).
